# Nationwide Shortage of Tuberculin Skin Test Antigens: CDC Recommendations for Patient Care and Public Health Practice

**DOI:** 10.15585/mmwr.mm6824a4

**Published:** 2019-06-21

CDC is expecting a 3–10 month nationwide shortage of Aplisol, a product of Par Pharmaceuticals, and one of two purified-protein derivative (PPD) tuberculin antigens licensed by the Food and Drug Administration (FDA) for use in performing tuberculin skin tests. This time frame is the manufacturer’s current estimate and is subject to change. The manufacturer notified CDC that they anticipate an interruption of supply of Aplisol 5 mL (50 multidose vials) beginning in June 2019, followed by an interruption of the supply of Aplisol 1 mL (10 multidose vials) in November 2019. The expected shortage of Aplisol 1 mL could occur before November 2019 if demand increases before then. Information on the status of this supply interruption will be updated at FDA’s Center for Biologics Evaluation and Research–Regulated Products: Current Shortages website (https://www.fda.gov/vaccines-blood-biologics/safety-availability-biologics/cber-regulated-products-current-shortages). This report includes CDC recommendations for mitigating a reduction in tuberculosis (TB) testing capability resulting from the anticipated Aplisol shortage (*1*).

Two types of immunological methods (tuberculin skin tests [TSTs] and interferon-gamma release assay [IGRA] blood tests) are used for detecting *Mycobacterium tuberculosis* infection. TSTs and IGRAs are used for the diagnosis of latent TB infection and can aid in the diagnosis of TB disease, but additional evaluation and testing is necessary to distinguish between latent TB infection and TB disease to determine the appropriate treatment (*2*). When findings such as chest radiography and mycobacterial cultures are sufficient for confirming or excluding a TB diagnosis, the results from a TST or an IGRA blood test might not be needed (*2*). However, most TB cases in the United States are diagnosed through a combination of findings, including results from one of these tests. When TB disease is strongly suspected, specific treatment should be initiated, regardless of results from TST or an IGRA blood test (*3*,*4*).

Two FDA-approved PPD tuberculin antigen products are available in the United States for use in performing TSTs: Tubersol (Sanofi-Pasteur) and Aplisol. In controlled studies, the concordance between the two products is high (*5*).

## Recommendations

CDC recommends the following three general approaches to mitigate a reduction in TB testing capability resulting from the expected shortage of Aplisol:

• Substitute IGRA blood tests for TSTs. Clinicians who use the IGRA blood tests should be aware that the criteria for test interpretation are different from the criteria for interpreting TSTs (*3*).

• Substitute Tubersol for Aplisol for skin testing. In studies, the two skin test products give similar results for most patients (*5*).

• Prioritize allocation of TSTs, in consultation with state and local public health authorities. Prioritization might require the deferment of testing some persons. CDC recommends testing only for persons who are at risk for TB (*6*–*8*). Groups at high risk for TB infection include 1) persons who are recent contacts exposed to persons with TB disease; 2) those born in or who frequently travel to countries where TB disease is common; 3) those who currently or previously lived in large group settings (such as homeless shelters or correctional facilities); 4) persons with compromised immune systems, including those with health conditions or taking medications that might alter immunity; and 5) children, especially those aged <5 years, if they are in one of the risk groups noted above.

Although overall test concordance is high, switching between PPD skin test products or TSTs and blood tests in serial testing might result in apparent conversions from negative to positive or reversions from positive to negative that might be attributable to inherent interproduct or intermethod discordance rather than change in *M. tuberculosis* infection status (*3*,*9*). Clinicians should assess test results based on the person’s likelihood of infection and risk for progression to TB disease, if infected (*2*).

In settings with a low likelihood of TB exposure, the deferment of routine serial testing should be considered in consultation with public health and occupational health authorities. Annual TB testing of health care personnel is not recommended unless there is a known exposure or ongoing transmission (*9*).
